# The London Water Supply

**Published:** 1896-11-07

**Authors:** 


### The Hospital

A Journal of

The Medical Sciences and Hospital Administration, with

AN EXTRA NURSING SUPPLEMENT.

Vol. XXI.—No. 528.] [Nov. 7, 1896.

### The London Water Supply.

Attention has recently been strongly directed to the condition of the water supply of London by a series of reports presented to the London County Council on the subject; reports, the tenor of which has been largely subversive of those assurances of safety which are continually published by the water companies, and were practically endorsed by the Royal Commission of 1893. It is well known that the conclusions of that Royal Commission, much as they have been quoted in defence of the present condition of affairs, have always been regarded with grave suspicion by scientific men, and it is a matter of the most serious importance that, as the result of further investigations made by men who are all eminent in their own departments, we should have presented to us in the same year (1) a report by Mr. Shirley Murphy, the medical officer of health, showing the great probability that much of the typhoid fever which we have in London (which is fortunately no great amount) is due to the water; (2) a report from Mr. Dibdin, chemist to the County Council, giving actual quantitative determinations and analyses of the "suspended matters" which have again and again been said not to exist, but of the presence of which he gave evidence before the Royal Commission, and showing by microscopical examination that this suspended matter contained such objectionable ingredients as "microscopic eels or anguillulæ, &c., decaying *débris* infested with bacteria, the larger infusoria, such as paramæcium, rotifera, &c., and epithelial scales"; (3) a report by Dr. Dupré confirming these results, and stating that "the analyses prove that the rivers Thames and Lea are subjected to pollution by sewage and surface drainage, for the complete removal of which the purifying power of the rivers themselves and the artificial filtration have been insufficient"; (4) a report by Dr. Klein, showing the frequent presence in the water supplied by various companies of large numbers of animalculi, in some cases more than a hundred times as large as the largest bacteria. Not only does the presence of these animalculi, some of which are clearly derived from sewage, show the foul origin of the water, but they, in even greater degree, demonstrate the imperfection of the filtration to which it has been subjected, so that it is not surprising to find that in a large number of instances the bacillus coli was "numerously present"; (5) a report by Dr. Stevenson in which he says, "Broadly, I may state that on one day in three the companies supplied inefficiently filtered water."

The importance of these reports is evident enough. The Royal Commission said, "We are strongly of opinion that the water as supplied to the consumer is of a very high standard of excellence and purity, and that it is suitable in quality for all household purposes," and now we are not only told that it is open to grave suspicion of distributing fever, but that it actually does, as shown by weights and measures, distribute many tons per annum of Thames mud. The probability of a direct connection between the water supply and certain diseases is made all the greater by a recognition of the fact that the so-called "filtered" supplies frequently contained minute entozoic worms, textile fibres, such as wool, cotton, and silk, and "fragments of matter in a state of decomposition, and swarming with various bacteria," and that among the living organisms frequently found were the bacterium coli and the paramæcium coli, one of which is a normal inhabitant of the human intestine, and the other "an almost normal inhabitant of fæcal matters." So much for the disgusting side of the accusation brought against the London water. Much emphasis has been laid upon this side of the question by various writers on the subject; but the other side of the accusation, viz., that the companies could have prevented this foulness if they had chosen so to do, while even emphasising still more the culpability of the companies in the past, holds out great hopes for the future in regard to the possibility of being able with proper care to obtain a pure supply from our present sources.

Everyone admits that at times the water supplied by the companies is very well filtered, and is practically pure. What is charged is that these periods of good supply are frequently interrupted by periods in which the water is obviously imperfectly filtered. Filtration should, at least, remove suspended matter, yet the tables given in the report we refer to show the enormous variations which occur from time to time in the state of the water supplied by many of the companies in this regard. All the time, however, one particular company, which, like the others, draws from the Thames, goes on with hardly a variation. As in all the waters, an improvement took place during the course of last year; but with this company there were no ups and downs—the standard was maintained uninfluenced by the condition of the river.

If the West Middlesex can do this, why cannot the others do the same? We do not say that the West Middlesex supplied a pure water; sometimes its water was beaten by that of other companies, but, at least, its condition as regards suspended matter was steady, and it is this steadiness, this independence of the state of the river, at the particular standard of excellence which the company chose to adopt, that points to the possibility of getting pure water from the Thames.

Schemes which can only promise good water as a general thing, interspersed with intervals of filth, are entirely out of court. But the real drift of Mr. Dibdin's report, condemnatery as it is of the present state of affairs, is that with proper care in the filtering of the water, and with proper arrangements for the softening of it, good water can yet be got from the rivers.

